# The diagnostic value of metagenomic next-generation sequencing for identifying *Pneumocystis jirovecii* infection in non-HIV immunocompromised patients

**DOI:** 10.3389/fcimb.2022.1026739

**Published:** 2022-10-27

**Authors:** Mengyi Zhao, Ruiming Yue, Xiaoxiao Wu, Zhan Gao, Miao He, Lingai Pan

**Affiliations:** ^1^ Institute of Blood Transfusion, Chinese Academy of Medical Sciences, Chengdu, China; ^2^ Department of Critical Care Medicine, Sichuan Provincial People’s Hospital, University of Electronic Science and Technology of China, Chengdu, China; ^3^ Chinese Academy of Sciences Sichuan Translational Medicine Research Hospital, Chengdu, China

**Keywords:** metagenomic next-generation sequencing (mNGS), *Pneumocystis jirovecii (P. jirovecii)*, infection, transplantation, immunocompromised

## Abstract

**Background:**

*Pneumocystis jirovecii* pneumonia (PJP) remains an important cause of morbidity and mortality in non-HIV immunocompromised patients especially in transplant recipients. But its diagnosis remains challenging due to the insuffificient performance of conventional methods for diagnosing *Pneumocystis jirovecii(P. jirovecii)* infection. Therefore, the auxiliary diagnostic function of metagenomics next-generation sequencing (mNGS) in clinical practice is worth of exploring.

**Method:**

34 non-HIV immunocompromised patients who were diagnosed as PJP by clinical manifestations, imaging findings, immune status of the host, and Methenamine silver staining were tested by mNGS from October 2018 to December 2020 in Sichuan Provincial People’s Hospital. The clinical performances of mNGS for *P. jirovecii* infection diagnosis were also evaluated with genome reads abundance and comparing with other traditional diagnostic methods.

**Results:**

We diagnosed a total of 34 non-HIV PJP patients by the clinical composite diagnosis. Our data shows that, compared with the clinical microbiological test, the detection rate of mNGS for *P. jirovecii* in non-HIV infected PJP patients is significantly higher than that of Methenamine silver staining and serum 1-3-β-D-glucan. mNGS can be used as an auxiliary diagnostic tool to help diagnosis. The number of reads mapped to the genome of *P. jirovecii* and the duration of patients from onset to sampling collection were statistically significant between the two groups (Reads>100 and Reads ≤ 100) (8days *vs*. 23days, *p*=0.020). In addition, univariate analysis showed that C-reactive protein (15.8mg/L *vs*.79.56mg/L, *p*=0.016), lactate dehydrogenase (696U/l *vs*. 494U/l, *p*=0.030) and procalcitonin (0.09ng/ml *vs*. 0.59ng/ml, *p*=0.028) was also statistically significant between the two groups.

**Conclusions:**

An effective detection rate was achieved in PJP patients using mNGS testing of bronchoalveolar lavage fluid (BALF) or blood. The study also confirmed that the abundance of reads of *P. jirovecii* is related to the interval between the onset and sample collection. And the inflammation status during simultaneous mNGS detection might determine the abundance of pathogens. Hence, we conclude that the mNGS strategy could benefit disease diagnosis as well as treatment when complicated clinical infections appeared.

## Introduction

*Pneumocystis jirovecii* pneumonia (PJP) is a common opportunistic infection in the immunocompromised population (Eddens and Kolls, 2015). PJP cases have been reported frequently in solid organ transplant recipients, particularly in renal transplant recipients (Yiannakis and Boswell, 2016; Chen et al., 2020; Le Gal et al., 2020), and other patients like cancer, patients with congenital or acquired immunodeficiency or patients treated with immunosuppressive drugs, and so on (Wickramasekaran et al., 2017; Chen et al., 2020). With the progress of medical technology, the population of organ transplantation or immunosuppression is gradually increasing, so the population of PJP may also increase. Compared with the human immunodeficiency virus (HIV)-PJP, the early clinical symptoms of non-HIV PJP are atypical and nonspecific, and more likely to lead to alveolar damage and respiratory failure, and rapidly develop into severe pneumonia, resulting in extremely high mortality. (Monnet et al., 2008; Tasaka et al., 2010; Roux et al., 2014). At present, the incidence of PJP in the non-HIV population continues to increase, which deserves attention (Cillóniz et al., 2019).

Rapid pathogen diagnosis and accurate treatment play a key role in improving outcomes in non-HIV patients with PJP (Zhang et al., 2021). Since *P. jirovecii* cannot grow stably *in vitro*, the detection tools for *P.jirovecii*, such as microscopy and Gomori’s methenamine silver staining, have limitations with low positive rate. Because these traditional methods require a high pathogen burden in the lungs and experienced microbiologists to ensure microscopic detection rates of *P. jirovecii*, which may lead to a certain false negative (Ma et al., 2018; Le Gal et al., 2020; Jiang et al., 2021). Besides, real-time PCR based diagnostic tests have significantly improved sensitivity and specificity, while commercial kits have not yet been widely used in clinical in China (Summah et al., 2013; Zhang et al., 2021). Metagenomic next-generation sequencing (mNGS) can complement traditional diagnostic methods through high-throughput sequencing, which can directly detect nucleic acids from pathogens in clinical samples, and then analyze nucleic acid sequences through bioinformatics methods. Comparing with traditional methods, mNGS has a higher detection rate of PJP (Wang et al., 2019; Zhang Y et al., 2019; Irinyi et al., 2020; Li et al., 2020). In addition, compared with other traditional diagnostic methods, the turnaround time of mNGS would be accomplished within 48 hours, therefore, mNGS can play an advantage in clinically auxiliary diagnosis (Somasekar et al., 2017; Chiu and Miller, 2019). mNGS could simultaneously identify bacteria, fungi, viruses, and various parasites from clinical samples such as cerebrospinal fluid, plasma, tissue, pleural fluid, etc. in an unbiased, synchronized, and straightforward manner (Yao et al., 2016; Chen et al., 2021). In recent years, the feasibility of mNGS in the pathogen identifications from clinical samples has been confirmed (Miao et al., 2018).There are some kinds of pathogens, such as fungi or viruses are not detectable by conventional culture methods, but can be detected by mNGS accurately and showed a higher positive detection rate (Charpentier et al., 2017; De La Cruz and Silveira, 2017). Therefore, nowadays, the implementation of mNGS becomes a very important detection method for severe or emerging pathogen infection.

To validate our main hypothesis that mNGS would perform better in emerging infection agents identification, in this study, the clinical data, routine biochemical tests, microbial culture, and mNGS results of 34 non-HIV immunocompromised PJP patients admitted to Sichuan Provincial People’s Hospital were analyzed to evaluate the application value of mNGS in clinical diagnosis.

## Materials and methods

### Study population and specimen collection

We included patients who were highly suspicious of PJP from 2018 to 2020 in Sichuan Provincial People’s Hospital. At present, the reference standard for diagnosis of PJP is still mainly to find characteristic cysts and trophozoites through staining and microscopic examination of clinical specimens, combined with the immune status of the host, degree of immunosuppression, imaging characteristics, and other biochemical indicators are taken as the auxiliary diagnosis of PJP. In this study, we combined the above mentioned factors as the reference standard for diagnosing PJP. We didn’t conduct PCR, because this detection method is not commonly used in most clinical microbiology laboratories in China (Bandt and Monecke, 2007). Patients were eligible for enrollment if they met all the following criteria:1) Immunocompromised, such as hematological malignancies, solid organ transplantation, hematopoietic stem cell transplantation, rheumatic immune system diseases, long-term use of corticosteroids or immunosuppressants, skin system diseases, etc.; 2) Typical clinical manifestations include subacute attacks, progressive dyspnea, accompanied by symptoms such as fever, dry cough, dyspnea and fatigue, and progressive hypoxemia; 3) Highresolution chest CT showed typical diffuse reticular nodules in both lungs, beginning with hilar nodular interstitial infiltration, mainly diffuse ground-glass opacities, with occasional plaques and consolidations; ([Chinese guidelines for diagnosis and treatment of HIV/AIDS (2018)], 2018; Fishman and Gans, 2019; Wang et al., 2019). 34 immunocompromised patients were diagnosed as PJP based on the host clinical status and immune states, clinical features, imaging findings, the results of Methenamine silver staining, the result of mNGS, and the comprehensive judgment of two senior clinicians in the hospital.

The flow diagram of the study is described in **Supplemental Figure S1**. Clinical data of all confirmed cases were recorded, including demographic characteristics, sample collection, and clinical microbiological test results. After admission, bronchoalveolar lavage fluid (BALF) or blood was sent for mNGS test.

### Plasma sample: Sample processing and DNA extraction

The volume of 3-4 mL of whole blood was drawn from patients, placed in the blood collection tube(BD Biosciences, State of New Jersey, USA) and stored at room temperature for 3-5 minutes before plasma separation and centrifuged at 4,000 rpm for 10 min at 4°C(Eppendorf, Hamburg, Germany)within 8 h of collection. Plasma samples were transferred to new sterile tubes(Gene Era Biotech, California, USA).DNA was extracted from 300 uL of plasma using the TIANamp Micro DNA Kit (DP316, TIANGEN BIOTECH, Beijing, China) following the manufacturer’s operational manual. The extracted DNA specimens were used for the construction of DNA libraries (Long et al., 2016).

### Body fluid sample: Sample processing and DNA extraction

1.5-3mL BALF sample from the patient was collected according to standard procedures. 1.5mL microcentrifuge tube(Gene Era Biotech, California, USA) with 0.6mL sample and 1g 0.5mm glass bead were attached to a horizontal platform on a vortex mixer(Thermo, Massachusetts, USA) and agitated vigorously at 2800-3200 rpm for 30 min. 0.3mL sample was separated into a new 1.5mL microcentrifuge tube and DNA was extracted using the TIANamp Micro DNA Kit (DP316, TIANGEN BIOTECH) according to the manufacturer’s recommendation.

### Construction of DNA libraries

Then, the DNA library was constructed by DNA fragmentation, end repair of blunt ends caused by fragmentation, ligation of adapters for amplification and sequencing, and PCR amplification to enrich the target fragments using the MGIEasy Cell-free DNA Library Prep Set (MGI Tech, Shenzhen, China), according to the instructions of the manual. Agilent 2100 was used for quality control of the DNA libraries. Quality qualified libraries were sequenced by the BGISEQ-50/MGISEQ-2000 platform (Jeon et al., 2014).

### Sequencing and bioinformatics analysis

High-quality sequencing data were generated by removing low-quality reads, followed by computational subtraction of human host sequences mapped to the human reference genome (Hg19) using Burrows-Wheeler Alignment (Li and Durbin, 2009). The remaining data by removal of low-complexity reads were classified by simultaneously aligning to four Microbial Genome Databases, consisting of bacteria, fungi, viruses, and parasites.

The classification reference databases were downloaded from NCBI (ftp://ftp.ncbi.nlm.nih.gov/genomes/). Reference sequences contained 4,945 whole genome sequences of virus, 6,350 bacterial genomes or scaffolds, 1064 fungi related to human infection, and 234 parasites associated with human diseases. The Genbank accession number of *P. jirovecii* reference genome sequences using for the mapping is GCA_001477535.1.

### Detection methods for methenamine silver staining and serum 1-3-β-D-glucan

BALF and blood were smeared on glass slides. After drying naturally, the samples were stained with silver staining solution (Zhuhai beso Biotechnology Co., Ltd., Shenzhen, China) according to the operation instructions. After waiting 3-5 min, *P. jirovecii* was microscopically examined for cyst structure.

The cell wall components of fungi(1-3-β-D-glucan) were detected according to the instructions of the fungal (1-3)-β-D-glucan detection (chromogenic method) kit (Dana Biotechnology Co., Ltd., Tianjin, China).

### Statistical methods

All continuous variables were expressed as medians, the Mann-Whitney U test was used to compare the differences of continuous variables between the two groups, continuous variables with a *P* value 0.05 were considered statistically significant, and all tests were two-tailed. All statistical analyses were performed using SPSS 22.0 software.

## Results

### Patient demographics and clinical characteristics

In this study, *P. jirovecii* was identified in the BALF/blood of 34 patients by mNGS, who were diagnosed as PJP. Of these confirmed cases, 22 were male (64.7%) and 12 were female (35.3%). The mean age was 51.79 years (from 20 years to 84 years), 19 were kidney recipients, 1 liver recipient, 5 cases of connective tissue disease, 4 cases of blood system diseases, and 5 cases of others (including tumors and skin diseases). The detection results of sample types, the number reads of *P. jirovecii*, 1-3-β-D-glucan, and Methenamine silver staining are shown in **Table 1**. The patient had no significant adverse events following imaging studies, mNGS, and other diagnostic procedures.

**Table 1:** Clinical microbiology results, sequencing information and demographic information of *P. jirovecii* positive patients.

Patient	Sex	Sample type	mNGS reads	Methenamine silver staining	1-3-β-D-glucan (pg/mL)	diagnosis
P1	M	BALF	162	–	683.2	kidney recipient
P2	M	BALF	217	–	10	others
P3	M	BALF	21745	–	10	blood system diseases
P4	F	BALF	20206	–	10	kidney recipient
P5	M	BALF	39	–	10	liver recipient
P6	M	BALF	12287	–	220.6	kidney recipient
P7	M	BALF	239032	–	10	kidney recipient
P8	M	Blood	13	–	118.6	kidney recipient
P9	F	BALFBlood	2169	–	158.2	kidney recipient
P10	F	Blood	131	–	156.8	kidney recipient
P11	F	Blood	378	–	202.9	kidney recipient
P12	M	BALF	1712	–	205.2	kidney recipient
P13	F	BALF	2754	–	877.5	kidney recipient
P14	M	BALF	3265	–	169.9	others
P15	F	BALF	16562	–	214.6	kidney recipient
P16	F	BALF	339	–	10	connective tissue disease
P17	F	BALFBlood	758	–	214.9	others
P18	M	BALF	60	–	234.4	kidney recipient
P19	M	BALF	3366	–	120.2	blood system diseases
P20	M	BALF	3070	–	263.5	kidney recipient
P21	M	BALF	15973	–	101.7	connective tissue disease
P22	M	BALF	8876	–	10	others
P23	M	BALF	2579	+	70.6	blood system diseases
P24	M	BALF	32447	–	10	kidney recipient
P25	M	BALF	38419	–	270.9	kidney recipient
P26	M	BALF	780	–	903.3	kidney recipient
P27	F	BALF	48	–	235.8	kidney recipient
P28	F	BALF	32	–	197.6	connective tissue disease
P29	M	BALF	2534	–	274.4	blood system diseases
P30	F	BALF	1808	–	467.1	connective tissue disease
P31	F	BALF	10	–	10	connective tissue disease
P32	M	BALF	2814	–	54.8	kidney recipient
P33	M	BALF	1850	–	287.1	kidney recipient
P34	M	BALF	22	–	10	others

M male, F female, ND not done, –negative, + positive, 1-3-β-D-glucan reference range:0-60.

### mNGS information and related influencing factors

Among 34 patients who were diagnosed with PJP by mNGS, the number of reads mapped to *P. jirovecii* genome ranged from10 to 239032 and the mean number of reads for *P. jirovecii* was 2534. BALF/blood samples for mNGS were collected from 1 to 159 days after suspected patient infection, with a median collection time of 16.5 days, and it was found that there was no statistical difference. Based on the number of reads of *P. jirovecii*, we divided 34 patients into 2 groups, one group with reads ≤ 100(7 patients, 20.59%)and the other with reads>100(27 patients, 79.41%). Comparing the two groups, the duration from onset to sampling collection was generally shorter in patients with reads>100 than in those with reads ≤100 (8 days *vs*. 23 days, *p*=0.020). There was a statistically significant difference in the symptom of cough (7 persons *vs*. 14 persons, *p*=0.021),the symptom of sputum(5 persons *vs*. 7 persons, *p*=0.027), and clinical indicators such as C-reactive protein (15.8mg/L *vs*. 79.56mg/L, *p*=0.016), lactate dehydrogenase(696U/l, 494U/l, *p*=0.030) and procalcitonin (0.09ng/ml *vs*. 0.59ng/ml, *p*= 0.028) (**Table 2**).

**Table 2:** Comparison of different *P. jirovecii* reads abundance groups.

Item	mNGS reads ≤ 100 (N = 7)	mNGS reads >100 (N = 27)	P
Age (year)	55 (35,84)	49 (20,82)	0.565
Sex (female/male)	3/4	9/18	0.653
Fever (n/%)	4/57.14	19/70.37	0.511
Cough	7/100	14/51.85	0.021
Sputum	5/71.43	7/25.93	0.027
Dyspnea	2/28.57	13/48.15	0.360
Hemoptysis	0/0	1/3.7	0.611
Laboratory tests			
White blood cells (×10^9^/L)	7.75 (2.75,14.76)	9.48 (1.53,16.76)	0.949
Hemoglobin (g/L)	114 (63,141)	94 (58,142)	0.130
Platelet (×10^9^/L)	130 (111,351)	178 (23,487)	0.949
C-reactive protein (mg/L)	15.8 (3.09,74.8)	79.56 (4.28,227.3)	0.016
LDH (U/l)	696 (525,1110)	494 (0,1443)	0.030
PCT (ng/ml)	0.09 (0.05,5.54)	0.59 (0.05,28.74)	0.028
Duration from onset to BALF collection (days)	23 (11,37)	8 (1,159)	0.02

### Comparison of diagnostic value of mNGS, methenamine silver staining, and serum 1-3-β-D-glucan for PJP

Methenamine silver staining is the most commonly used clinical test for PJP, and serum 1-3-β-D-glucan is a widely used serological marker for PJP, if the value of 1-3-β-D-glucan is elevated, the patient may be infected with *P. jirovecii*. In the present study, we compared the diagnostic value of the mNGS, Methenamine silver staining and serum 1-3-β-D-glucan in 34 PJP patients. *P. jirovecii* was detected from patients by clinical microbiological testing methods, of which 34 patients were detected by Methenamine silver staining, only 1 was positive (2.9%) and 34 patients were detected by 1-3-β-D-glucan, and the value greater than 60 pg/mL was 67.65% (23/34). However, we detected *P. jirovecii* in 34 patients (100%) by mNGS. Thus the study shows that mNGS has a high detection rate for *P. jirovecii* infections, which also confirmed that the clinical mNGS performance is helpful for infection diagnosis.

## Discussion

Currently, the incidence of PJP in non-HIV immunocompromised populations is increasing due to the prevalence of immunosuppressive diseases such as hematological malignancies, solid tumors, systemic corticosteroid therapy, immunosuppressive therapy, and organ transplantation (Gaborit et al., 2019). It has also been shown that in non-HIV-infected people, PJP usually develops into respiratory failure within a short period and results in a 30–60% mortality rate, which is significantly higher than in HIV-infected people (Cillóniz et al., 2019). Therefore, the early diagnosis of *P. jirovecii* infections in non-HIV patients is particularly important. In the past, the diagnosis of *P. jirovecii* was mainly based on staining, PCR, and detection of 1-3-β-D-glucan is a common cell wall constituent of most pathogenic fungi, including *P. jirovecii* (Lu et al., 2011a). But these methods have their limitations. Routine staining requires a large number of pathogens in the lungs and an experienced microbiologist to ensure the detection of *P. jirovecii* under the microscope; therefore, it can be insensitive and biased (Procop et al., 2004). Studies have demonstrated that respiratory specimen PCR results are sufficient to confirm or rule out disease in high-risk patients with suspected *P. jirovecii* (Lu et al., 2011b), but it might fail value in single-shot detection of mixed infections, especially for those rare or novel strains (White et al., 2017). In addition, PCR and microarrays use specific primers or probes to target only one or a limited number of known pathogens, which is very inconvenient. However, for all DNA or RNA present in the sample, mNGS allows detection of the entire microbiome and host genome or transcriptome in patient samples (Camargo et al., 2019; Wang et al., 2019; Irinyi et al., 2020; Li et al., 2020). Besides, the non-specificity of serum 1-3-β-D-glucan limits its application in the diagnosis of PJP (Del Corpo et al., 2020). 1-3-β-D-glucan is a common cell wall constituent of most pathogenic fungi, including *P. jirovecii* (Lu et al., 2011a; Li et al., 2015). The 1-3-β-D-glucan assay has been approved for making a diagnosis of invasive fungal disease (De Pauw et al., 2008). However, the role of the serum-1-3-β-D-glucan assay in the diagnosis of PJP is controversial, especially among patients with or without HIV infections (Li et al., 2015). Studies have demonstrated that a negative result of the serum-1-3-β-D-glucan determination is sufficient to rule out PJP in HIV cases only, but in non-HIV patients, 1-3-β-D-glucan assays are insensitive and nonspecific for invasive fungal disease (Li et al., 2015; Del Corpo et al., 2020), thus, the results should therefore be carefully analyzed in parallel with clinical features, radiological findings, and other diagnostic evidence (Lu et al., 2011a; Li et al., 2015; Del Corpo et al., 2020). In conclusion, these methods will lead to the delay of clinical treatment and affect the prognosis of patients. Therefore, it is necessary to find a method with high diagnostic accuracy, short detection time, and accurate identification of infectious pathogens (Jiang et al., 2021). mNGS has an efficient workflow, relatively low-cost consumption, and short turnaround time. Hence mNGS may be widely accepted in clinical practice (Han et al., 2019). Moreover, mNGS also has the characteristics of high throughput and high sensitivity and can measure millions or even hundreds of millions of nucleic acid sequences at the same time, which plays a very important role in the accurate diagnosis of infectious diseases (Yu et al., 2011; Wilson et al., 2014; Ni et al., 2015; Tong et al., 2015).The diagnostic performance of mNGS in the respiratory tract (Langelier et al., 2018; Xie et al., 2019), bloodstream (Dubourg and Raoult, 2016), central nervous system (Ramachandran and Wilson, 2020), pleural cavity (Chen et al., 2021), and prosthetic joint infections (Thoendel et al., 2018) has been appreciated. Research data also supports its advantages in detecting opportunistic pathogens and co-infections, especially uncultivable pathogens (Parize et al., 2017; Pan et al., 2019), such as *Legionella pneumophila, Aspergillus* spp. (Yue et al., 2021)*, Nocardia* spp. (Ding et al., 2021)*, Mucor* spp. (Liu et al., 2021) etc.

In this study, clinical microbiology tests were compared with mNGS, and our data shows that the detection rate of mNGS for *P. jirovecii* in non-HIV infected PJP patients is significantly higher than that of serum 1-3-β-D-glucan and Methenamine silver staining, which can well auxiliary clinical diagnosis. Previous research shows that if the microorganism identified only by mNGS is accompanied by discrete evidence in clinical practice, the pathogen is considered a pathogen with a certain degree of clinical suspicion (Wu et al., 2020; Jiang et al., 2021). In our study, 34 patients with PJP were diagnosed through clinical comprehensive diagnosis and mNGS results, suggesting that mNGS plays an important role in assisting diagnosis of pathogens with a certain degree of clinical suspicion, which is consistent with the views in the above findings. We also analyzed the time interval from onset to the collection of samples and found that the interval time of the group with reads>100 was significantly shorter than that of the group with reads ≤ 100, which indicated that the number of reads detected by mNGS for *P. jirovecii* was closely related to the time of sample collection. This result is also consistent with previous studies, indicating that early treatment, effective use of antibiotics, and improved disease status will reduce the number of reads detected by mNGS for pathogens (Ai et al., 2018; Zhang Xx et al., 2019).

In addition, we compared some factors that may affect the reads abundance of *P. jirovecii* detected by mNGS. The results of the univariate analysis showed that age, gender, white blood cells, hemoglobin, and platelets did not affect the reads abundance of *P. jirovecii*, while C-reactive protein, LDH, and procalcitonin were significantly different between the group with reads>100 and the group with reads ≤ 100.These results suggest that concurrent detection of inflammatory status during mNGS may determine the pathogen abundance, which is also consistent with previous studies (Zhang Xx et al., 2019).

This study also has certain limitations. Firstly, due to the small sample size, the positive rate of clinical routine method evaluation is low, so the sensitivity of mNGS may be slightly overestimated which belongs to partial validation bias. Secondly, because BALF is adopted at different positions of lung segment, the partial deviation will occur, which will affect the reads abundance of *P. jirovecii* accompanied with the sensitivity evaluation. Thirdly, due to limited conditions, the hospital did not routinely perform PCR for *P. jirovecii* identification, so the diagnostic performance of mNGS and PCR was not compared. Finally, mNGShas disadvantages, for example, it is difficult for mNGS to distinguish *P. jirovecii* colonization from infection because there is no widely accepted mNGS quantification cutoff or threshold. In addition, the sequencing depth of mNGS is still limited, and the pathogen database needs further improvement (Gargis et al., 2016). Therefore, a definitive diagnosis of PJP must be based on a comprehensive summary of clinical features, laboratory abnormalities, imaging findings, and microbiological evidence, rather than mNGS alone (Jiang et al., 2021). However, the rapid development of next-generation sequencing technology will show higher sensitivity and specificity in diagnosing infections in the future (Thorburn et al., 2015), and we are confident that the mNGS will effectively give a valuable performance in clinical infection diagnosis.

## Data availability statement

The datasets presented in this study can be found in online repositories. The names of the repository/repositories and accession number(s) can be found below: https://bigd.big.ac.cn/gsa/browse/CRA008494 and the assigned accession of the submission is: CRA008494.

## Ethics statement

This study was reviewed and approved by Medical ethics committee of Sichuan Academy of Medical Sciences and Sichuan Provincial People’s Hospital. The patients/participants provided their written informed consent to participate in this study.

## Author contributions

All authors contributed to the study conception and design. MZ, RY, LP, and MH wrote the manuscript draft. RY, XW, and ZG conducted most of the experiments. MZ and MH performed data analysis. LP and MH conceived the idea and directed the experiments. All authors contributed to the article and approved the submitted version.

## Funding

This study was supported by Research and Development Projects of Science and Technology Department of Sichuan Province (Grant No.2019YFS0319), Sansure Biotech Transfusion Medicine Development Fund of Chinese Society of Blood Transfusion (CSBT-SX-2021-01) and Project of the Special Fund for Young and Middle aged Medical Research of the China International Medical Exchange Foundation(Z-2018-35-19202).

## Acknowledgments

We acknowledge Dr. Fan Zhenxin (College of Life Sciences, Sichuan University) for bioinformatics helping, and Mr. Wang Baoshan (Mountain in Sight (Sichuan) Medical Technology Co. Ltd) for coordinating sample collection and shipping.

## Conflict of interest

The authors declare that the research was conducted in the absence of any commercial or financial relationships that could be construed as a potential conflict of interest.

## Publisher’s note

All claims expressed in this article are solely those of the authors and do not necessarily represent those of their affiliated organizations, or those of the publisher, the editors and the reviewers. Any product that may be evaluated in this article, or claim that may be made by its manufacturer, is not guaranteed or endorsed by the publisher.
