# Supplementary figures and images for: The diagnostic value of metagenomic next-generation sequencing for identifying Pneumocystis jirovecii infection in non-HIV immunocompromised patients
Source: Front Cell Infect Microbiol. 2022 Oct 27;12:1026739. doi: 10.3389/fcimb.2022.1026739 (PMC9647189; doi:10.3389/fcimb.2022.1026739)

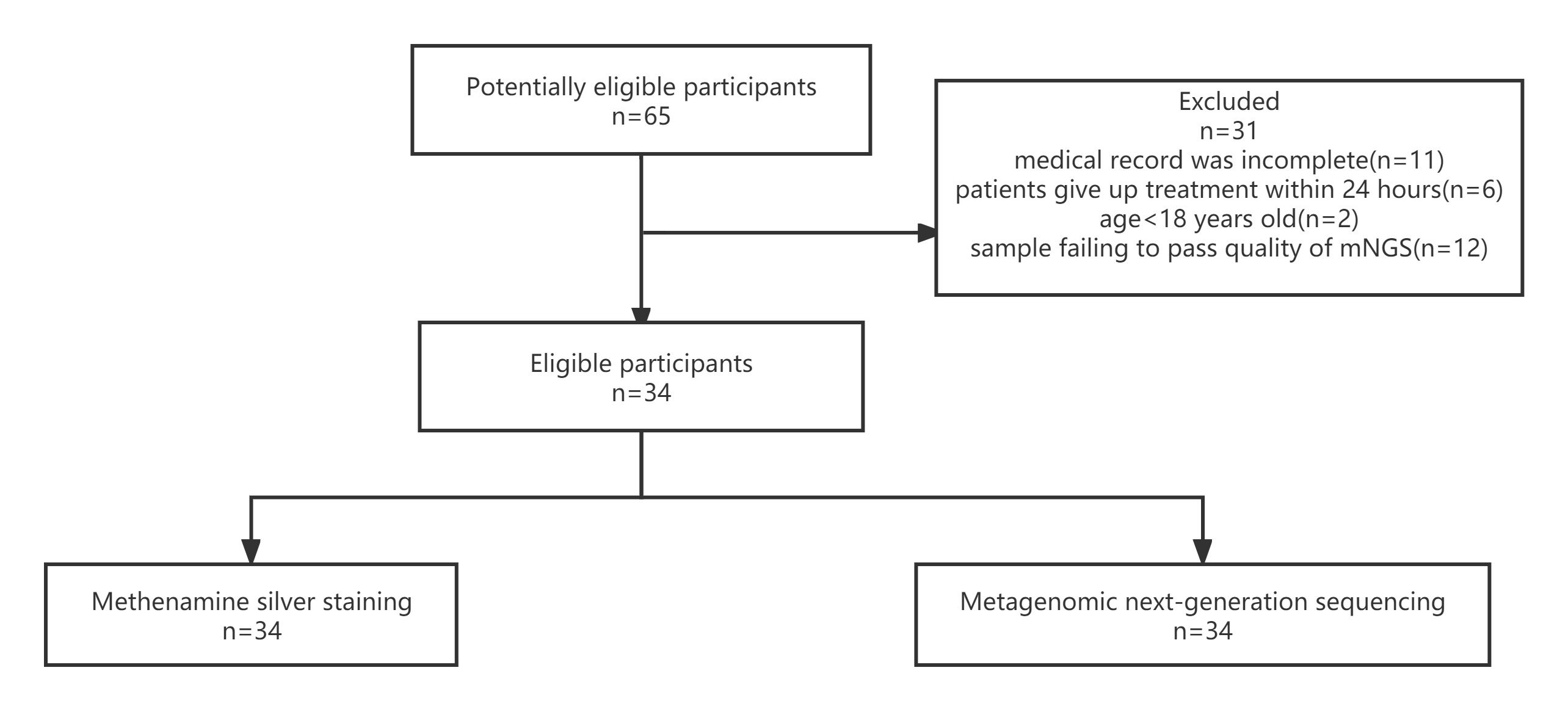

Supplement: Supplementary file 1 [file Image_1.jpg]
